# Bioactive Coating with Two-Layer Hierarchy of Relief Obtained by Sol-Gel Method with Shock Drying and Osteoblast Response of Its Structure

**DOI:** 10.3390/nano7100323

**Published:** 2017-10-13

**Authors:** Elena G. Zemtsova, Andrei Y. Arbenin, Natalia M. Yudintceva, Ruslan Z. Valiev, Evgeniy V. Orekhov, Vladimir M. Smirnov

**Affiliations:** 1 Saint Petersburg State University, Universitetskii pr.26, 198504 Saint Petersburg, Russia; rzvaliev@gmail.com (R.Z.V.); zeka@list.ru (E.V.O.); vms11@yandex.ru (V.M.S.); 2 Institute of Cytology of the Russian Academy of Sciences (RAS), Tikhoretsky ave., 4, 194064 Saint Petersburg, Russia; yudintceva@mail.ru

**Keywords:** composite, sol-gel, dip coating, two-level hierarchy of relief, bioactivity, osseointegration, implant, titania, osteoblast

## Abstract

In this work, we analyze the efficiency of the modification of the implant surface. This modification was reached by the formation of a two-level relief hierarchy by means of a sol-gel approach that included dip coating with subsequent shock drying. Using this method, we fabricated a nanoporous layer with micron-sized defects on the nanotitanium surface. The present work continues an earlier study by our group, wherein the effect of osteoblast-like cell adhesion acceleration was found. In the present paper, we give the results of more detailed evaluation of coating efficiency. Specifically, cytological analysis was performed that included the study of the marker levels of osteoblast-like cell differentiation. We found a significant increase in the activity of alkaline phosphatase at the initial incubation stage. This is very important for implantation, since such an effect assists the decrease in the induction time of implant engraftment. Moreover, osteopontin expression remains high for long expositions. This indicates a prolonged osteogenic effect in the coating. The results suggest the acceleration of the pre-implant area mineralization and, correspondingly, the potential use of the developed coatings for bone implantation.

## 1. Introduction

In recent decades, bone implantation has been actively developed. This procedure became possible after the discovery of osteointegration by Brånemark et al. [1]. Currently, metallic titanium and its alloys are widely used for the fabrication of bone and dental implants [2,3,4]. This can be explained by its good mechanical properties, and by its ability to be integrated into hard tissue. Taking into account all of the advantages of pure titanium, we should note, however, that there are some approaches to surface modification that have led to the enhancement and acceleration of its osteointegration [5,6,7,8]. Such an effect diminishes the engraftment duration. This effect has also resulted in the observed decrease of statistics related to implant loss due to rejection, loosening due to a weak contact, and infection. Currently, the main advantages of implant modification are known: micron-size implant roughness leads to significantly better engraftment, and better contact with the bone is achieved, which can be confirmed both by histological analysis and by biomechanical experiments [9,10,11]. Additionally, nanometer-sized relief has a positive influence on the osteointegration process [12,13]. This effect is especially significant during the process of osteoblast differentiation that correlates with the start of the mineralization of the peri-implant area [14]. Surfaces with two-level (micro and nano) relief hierarchies have a significant cytological and histological response [15,16]. The combination of two levels of relief organization in the surface layer of a single implant provides a significant synergistic effect on osteointegration, without the loss of individual contributions.

Usually, a two-level hierarchy is reached through a combination of techniques directed towards the formation of micro- and nanoreliefs. A rare exception is described in [17], where both kinds of roughness were achieved by the same laser treatment technique. Combination of acidic etching and cathodic sputtering [18] produced micron-sized pits and nanometer-sized titanium knots, respectively. According to cytological studies, such a surface structure leads to a significant increase in the implant’s bioactivity. Combination of double acidic etching with anodizing [15] led to the growth of nanotubes oriented normally to the surface. Acidic etching with subsequent alkaline treatment [17] produced so-called nest-shaped structures composed of micron-sized cells and nanometer-sized plates. The surfaces of these structures were very well developed. A similar approach was used in [16]: micron-sized roughness was reached by sandblasting, while nanosized roughness was achieved by alkaline treatment. This led to nanometer-sized titanium dioxide plates. One more interesting technique is presented in [19]. Nanorelief in the form of TiO_2_ nanotubes was achieved by anodizing. In contrast to the above mentioned works, here, micron-scale relief was obtained without erosion: titanium substrate was treated by titanium powder using vacuum plasma sputtering. This provided a well-developed micron-sized relief.

A comparative analysis of the contributions of micro- and nanorelief to the biomedical properties of the implant surface was performed in [20]. Here, pure titanium, along with micron-sized samples after etching and nanosized samples after anodization, was used for cytological studies. It was shown that both kinds of treatment led to a positive effect; however, their combination in the same sample provided highly pronounced synergy.

We have summarized the techniques for fabrication of two-level hierarchical coatings. All of them are based on pairs of techniques; one technique provides micron-sized relief and the second technique provides nanosized relief. Combinations could include

- sandblasting and anodizing,
- acidic etching and alkaline treatment,
- sol-gel synthesis and shock-drying,
- acidic etching and cathodic sputtering, etc.

In all works, a positive effect of modification was observed. It is worth noting that this approach is prominent not only due to its enhancement of biomedical parameters, but also due to the manufacturability of the modification methods.

In this work, we analyze the efficiency of implant surface modifications by means of a two-level relief hierarchy created by a sol-gel method with subsequent shock drying. Using this approach, we fabricated a nanoporous layer with micron-sized defects. This article continues our previous work [21], in which we showed the effect of acceleration of osteoblast-like cell adhesion after this kind of modification. More detailed evaluation of the efficiency was based on a deep cytological analysis that included the study of the marker levels of the osteoblast-like cell differentiation, meaning the mineralization of the peri-implant area. These coatings have good biomedical characteristics, while the classical methods used in the synthesis—sol-gel, immersion and heat treatment—make its execution rather easy, technologically speaking.

In this work, UFG-titanium was used as a substrate. The choice was based on its perfect mechanical properties (tensile strength—1240 MPa, fatigue endurance limit at 10^6^ cycles—590 MPa), which is very important for implantation due to the possibility of miniaturization of the implants and, consequently, the reduction of the wound surface.

## 2. Materials and Methods

### 2.1. Chemicals

Sigma-Aldrich reagents (St. Louis, MO, USA) were used: titanium (IV) isopropoxide (Ti(OCH(CH_3_)_2_)_4_, TTIP, 98%+), diethanolamine (HN(CH_2_CH_2_OH)_2_, DEA, 99%+), isopropanol ((CH_3_)_2_CHOH, 99.9%+).

As the substrate for coatings, ultrafine grained (UFG) titanium plates were used. These plates were obtained by means of severe plastic deformation at 400 °C in ECAP-Conform (Equal Channel Angular Pressing) mode with multistage surface polishing to a roughness less than R_z_ = 0.01 μm: this was performed mechanically on a Ecomet (Buehler, Lake Bluff, IL, USA) using various abrasives: P 320, P 600, P 1200. Subsequently, polishing was done using colloidal silica Mastermet. The samples were 5 mm × 25 mm in size.

Materials for the cytological studies: nutrient medium DMEM (Gibco, Waltham, MA, USA), fetal *bovine* serum (FBS, HyClone, Logan, UT, USA), mixture of antibiotics penicillin/streptomycin (Sigma-Aldrich, St. Louis, MO, USA), MC3T3-E1 osteoblast-like cell culture.

Cytological testing kits: Alkaline Phosphatase Assay Kit (Colorimetric) (Abcam, San-Francisco, CA, USA) for analysis of the alkaline phosphatase activity, Osteopontin N-Half ELISA Kit (Clon tech, Hamburg, Germany) for analysis of the osteopontin expression.

### 2.2. Fabrication of TiO_2_ Gel Films on the Titanium Surface

The sol-gel technique is very convenient both for nanolayers with thicknesses up to 100 nm, and for films with thicknesses from 100 nm to tens of micrometers of various oxide gels on the solid substrates. Note that the layer thickness can be controlled in two different ways. The first way is by varying the rate at which the plate is taken out of the solution. The second way is based on cycling the dip-coating process for the number of times necessary after the first layer coating.

TiO_2_-based film coating was carried out using KSV NIMA Dip Coater Single Vessel, KSV NIMA, Biolin Scientific, Västra Frölunda, Sweden. As the basis, the technique for growing TiO_2_ films on solid substrates by dip-coating from a non-aqueous medium, previously reported in [22], was used. Initially, TTIP and DEA were dissolved in anhydrous *i*-PrOH. After that, ca. twice the molar excess of water was slowly introduced, relative to the TTIP amount. This provided incomplete hydrolysis of the titanium compound. The final weight ratio of the components was TTIP/*i*-PrOH/DEA/H_2_O = 227/773/105/36.

As-prepared alcoholic solution of titanium polydiisopropoxide was used for the film precipitation in dip-coating mode: the substrate was taken out at a rate of 75 mm/min; cyclization of the process was used to increase the thickness. Note, especially, that the film synthesis was performed based on sol-gel technology. So, when applied to a substrate, the solution was initially converted to a sol and then to gel, because of the hydrolysis of titanium oxide precursor in the wet atmosphere.

### 2.3. Texturing of the TiO_2_ Gel Layers on the Nanotitanium Surfaces Based on Shock Drying

In order to increase the roughness of the TiO_2_ layer surface due to the appearance of defects, we used the shock-drying technique on a hot plate. The nanotitanium sample with the freshly precipitated TiO_2_ gel layer was placed onto the surface, which was pre-heated to 400 °C, for 10 min. This led to the rapid contraction of the film due to the loss of disperse media. The film contained crack-like defects. These defects are due to the tensions that appeared during contraction. These cracks had micron-size length, and sub-micron width; continuous film zones delineated by the cracks had a size of up to tens of microns.

### 2.4. Fabrication of the TiO_2_ Xerogel Film

As-precipitated films of the gel were not converted into xerogel even after short-time heat treatment at 400 °C. This was due to the presence of diethanolamine and the products of incomplete hydrolysis of titanium tetraisopropoxide in the pores. In order to obtain empty pores, we initially boiled the samples in distilled water. After that, the water was removed from the pores by calcining in air at 300 °C. Thus, xerogel films were obtained. As we showed in our earlier work [21], this method leads to anatase.

### 2.5. Sample Characterization

#### 2.5.1. Structure of the TiO_2_ Xerogel Layers

To evaluate the film thickness, we used spectral ellipsometric complex «Ellips 1891 SAG» with a wavelength range of 350–1000 nm, suitable for measuring multilayer coatings as well as porous coatings.

The structure and morphology were studied using a scanning electron microscope Merlin (Carl Zeiss Microscopy GmbH, Jena, Germany) and Atomic Force Microscope NTEGRA (NT-MDT, Moscow, Russia), allowing the evaluation of both the front view of the coating and its profile.

#### 2.5.2. Cytology of the Coatings

The ability of the cell line MC3T3-E1 to differentiate was controlled by measuring the change of alkaline phosphatase activity and osteopontin expression in the cells while incubating on the sample surfaces. Samples of the titanium matrices after preliminary ozonation were placed into a 6-hole culture plates (Nunc; Thermo Fisher Scientific, Waltham, MA, USA). MC3T3-E1 osteoblast-like cell suspension was seeded onto the samples of nanostructured titanium matrices. This suspension, at a concentration of 6 × 10^4^/cm^3^ in the cultural medium (nutrient medium DMEM (Gibco), contained 10% fetal bovine serum (FBS, HyClone) and a mixture of the antibiotics penicillin/streptomycin (Sigma-Aldrich). As the inductive medium for the differentiation, the DMEM medium was served with low glucose content (1 g/L), and the addition of dexamethasone (107 mM), R-glycerophosphate (10 mM), and ascorbic acid (0.2 mM) (Gibco). Incubation was performed over various durations (0, 7, 14, 21 days). After finishing, samples of the culture-conditioned medium were taken. MC3T3-E1 osteoblast-like cell were cultivated on the surface of culture plates and used as a control. Alkaline phosphatase activity was determined colorimetrically using Alkaline Phosphatase Assay Kit (Colorimetric) (Abcam). Osteopontin expression was determined colorimetrically using Osteopontin N-Half ELISA Kit (Clon tech). For each cell analysis, we used 5 samples, and performed the analysis 3 times. Statistical analysis of experimental data was performed using one-way analysis of variance (ANOVA) and a *p*-value less than 0.05 was considered statistically significant.

## 3. Results

According to the developed technique, we fabricated a series of shock-dried gel film samples of different thicknesses, generated as a result of variations in the number of dip-coating cycles. After 1–5 cycles of dip coating, the corresponding samples 1–5 were obtained. The relationship between thickness and number of coating cycles is given in Table 1.

**Figure 1:**
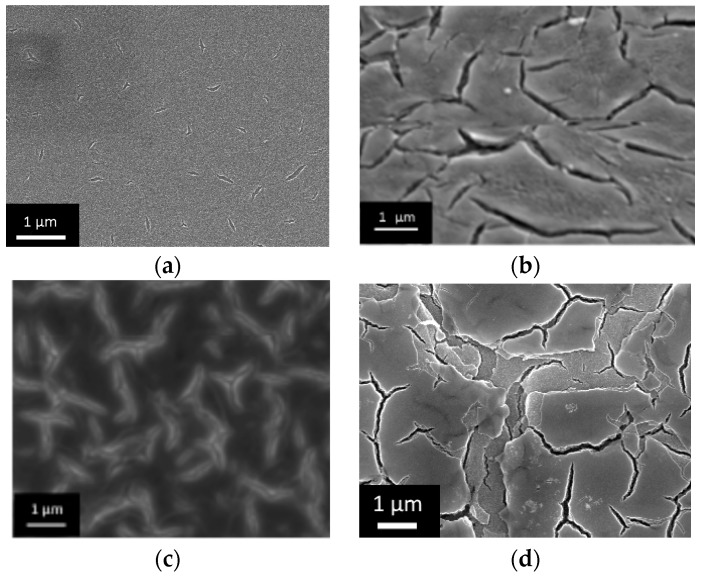
Electronic microphotographs of the textured TiO_2_ xerogel films of various thickness obtained by shock drying freshly precipitated gel. (**a**–**d**—2, 3, 4, 5 cycles of dip coating).

Sample 1 has small film thickness, and there are no defects—its structure corresponds to the published data [22]. Sample 2 with two layers has single discontinuities that serve as precursors to the cracks. Samples 3 and 4 have well-pronounced cracks. In sample 5, with 5 layers, cracks form the net. Zones of detachment appeared on the sections outlined by the cracks. Images of the films are shown in Figure 1a–d.

**Figure 2:**
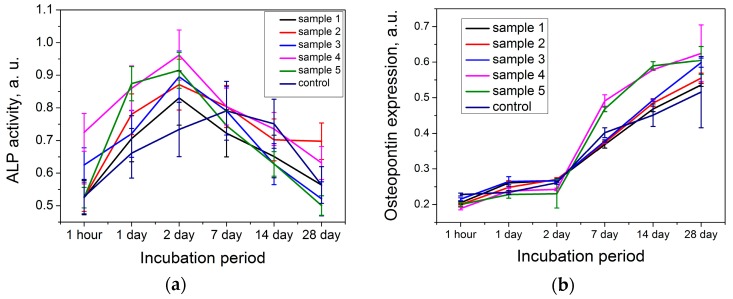
Assessment of expression of osteogenic markers: alkaline phosphatase activity (**a**) and osteopontin expression (**b**) of osteoblast-like cells incubated on the samples 1–5 surface as compared with the control sample.

Cytological study demonstrates an interesting effect of surface organization on the osteoblast-like cell behavior. We noted recently [21] that the samples with a developed relief demonstrate enhanced surface coverage by osteoblast-like cells and adhesion. In addition to this, we investigated the alkaline phosphatase activity and osteopontin expression that indicated the mineralization of the pre-implant area for short and long incubation periods. The change in the activity of the cells cultivated on the surface of the samples under study was different, when compared to the control (Figure 2). When the cells were cultivated on the surface of studied samples, the activity increased over 1 and 2 days. Sample No 4 exhibited early activity, which indicates acceleration of osteoblast-like cell differentiation. This effect could significantly influence the rate of implant engraftment, because acceleration of differentiation leads to the contraction of induction time before peri-implant area mineralization. It is interesting that less beneficial properties were observed for sample 5, despite this sample having analogous film structure at the nanometer scale, and more developed micron-sized relief compared to sample 4. This could be attributed to the fact that cracks aggregated to the net, and film elements delineated by cracks were exfoliated. This erosion negatively influences the formation of the cell layer. The dynamics of osteopontin accumulation during cultivation of the cell line MC3T3-E1 on the samples surface is presented in Figure 2. Osteopontin was found in all the samples of the medium conditioned by the cell line MC3T3-E1. In inductive medium conditions, osteopontin accumulation mainly started after 2–7 days of cell cultivation on the sample surface. The active process continued for 14–28 days. The values were highest for samples 4 and 5. This indicates the positive influence of long periods of incubation on the mineralization process.

Therefore, we can summarize that the coatings on sample 4 were optimal for the improvement of the biomedical properties of bone implants, due to the quicker start of osteoblast-like cell differentiation, and the highest alkaline phosphatase activity and osteopontin expression. This effect can be explained by the presence of a two-level hierarchy of the surface relief of the sample, with a developed network of micron cracks and nanometer globules of xerogel of titanium oxide without delamination of the film.

**Figure 3:**
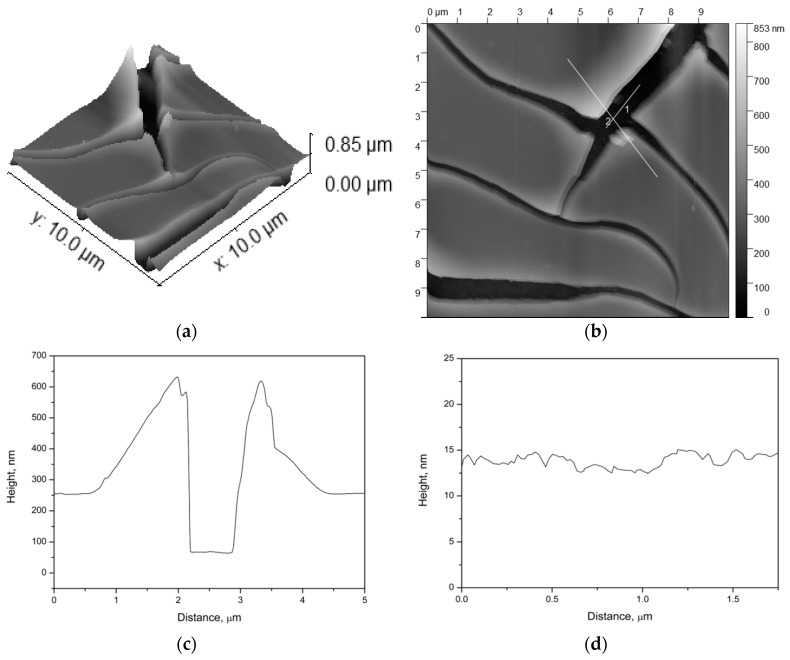
AFM image of the sample after 4 cycles of dip coating (**a**—3D model; **b**—frontal view with the lines of profile building; **c**—profile 1; **d**—profile 2).

In order to refine the structure of the best coating (sample 4), we performed an atomic force microscopy (AFM) study. AFM is able not only to reveal a frontal location of the cracks, but also to demonstrate the profile of the coated film (Figure 3).

**Figure 4:**
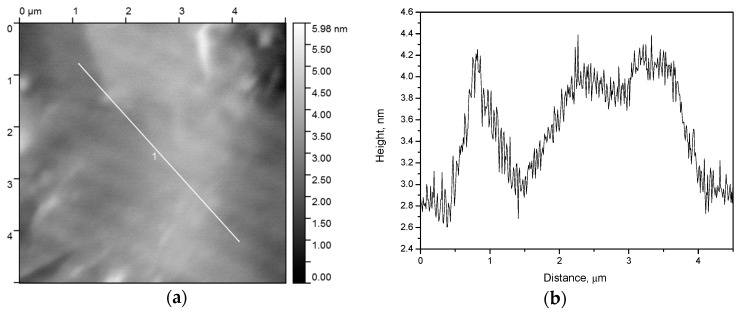
AFM image of the polished substrate with UFG titanium: **a**—front; **b**—slice by line 1 at previous picture.

As one can see (Figure 3), there are the cracks with the borders raised relative to the base film height, while inside the cracks, there is almost no xerogel. Slice 1 (Figure 3c) and the central part of slice 2 (Figure 3d) exhibit a plateau whose profile corresponds to the roughness of the initial substrate (Figure 4).

**Figure 5:**
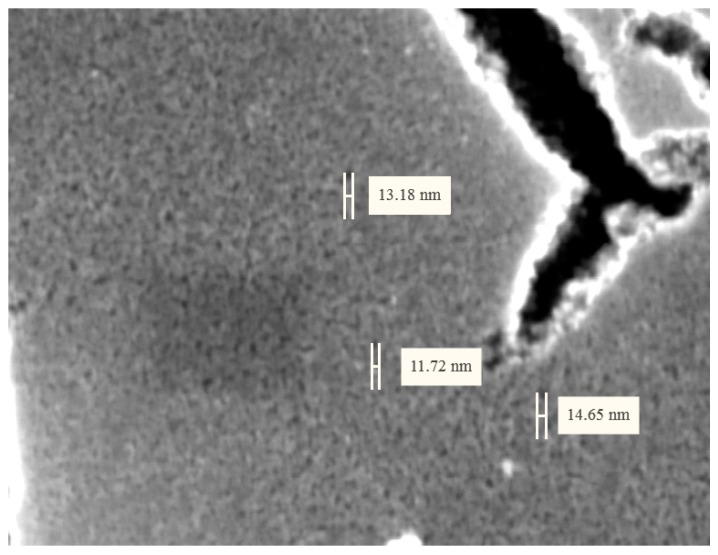
High-resolution electronic microphotograph of the sample after 4 cycles of dip coating.

Electronic microphotograph (Figure 5) indicates the presence of nanometer relief of the obtained coating, formed by globules of titanium dioxide xerogel.

Therefore, the optimal geometry was determined in sample 4, which was covered by a mesoporous (nanoglobular) film with submicron-size cracks that were not joined into a single net, and without delamination, despite sample 5.

## 4. Conclusions

We developed a technique for the synthesis of textured TiO_2_ xerogel films, based on the shock-drying of freshly precipitated gel films. The films have a two-level hierarchy of the surface relief due to nanometer-sized xerogel mesopores and the micron-sized structure of the defects. This surface layer ordering has a positive cytological response for osteoblast-like cells. These results correlate with the published data given at the beginning of the manuscript. Temperature of thermal treatment did not exceed 400 °C. Therefore, these coatings could be applied for the enhancement of biomedical properties of ultrafine grained titanium—a material that has a unique combination of mechanical properties [23] and biocompatibility. This makes it a very interesting prospective candidate for bone implantation. In summary, we found a new original approach for the development of bioactive coatings that allows improvement to the biomedical properties of bone implants.

## Figures and Tables

**Table 1:** Dependence of film thickness on the number of dip coating cycles.

Number of Cycles	1	2	3	4	5
Film thickness, nm	33 ± 1	72 ± 2	124 ± 2	173 ± 3	204 ± 4
